# Angiodysplasia in terminal ileum: Case report and review of literature

**DOI:** 10.1016/j.ijscr.2019.11.012

**Published:** 2019-12-16

**Authors:** Thamer Alghamdi

**Affiliations:** Albaha University, Faculty of Medicine, Surgery Department, Albaha Governorance, 1988, Saudi Arabia

**Keywords:** Angiodysplasia, Arteriovascular malformations, Ileal resection, Telangectasia

## Abstract

**Background:**

Angiodysplasia is the second most common vascular abnormality of the GI tract after diverticulosis and is the second principal cause of lower GI bleeding over 60 years.

**Case report:**

A 68-year-old male patient presented to our institute in the outpatient clinic with pallor of gradual onset and progressive course since 6 months. Detailed history revealed hematochezia, sometimes melena. Physical examination revealed signs of iron deficiency anemia which was confirmed by laboratory results and hemoccult positive stool. The patient refused the colonoscopy. A bdominal CT with contrast was then performed which revealed an evidence of abnormal subtle mural contrast flocculation within the small bowel loop with luminal extravasations of contrast at small segment of distal ileum few centimeters from ileocaecal valve with mildly dilated and early filling of its drained vein, this abnormal contrast extravasations and flocculation increased through dynamic study. In addition, evidence of contrast mural flocculation within the left lateral wall of distal few centimeters of rectum was also observed. This abnormal mural contrast flocculation within the wall of solitary segment terminal ileum and distal rectum suggest the evidence of venous ectasia or angiodysplasia. Surgical resection was done with end to end anastomosis of the small intestine without any complication in the postoperative progress.

**Conclusion:**

This case also emphasizes the critical significance of a mutual multidisciplinary approach to occult gastrointestinal bleeding. The management of angiodysplasia is considered as multidisciplinary team work that would consist of expertise from different departments as in endoscopy, angiography, and gastrointestinal surgery.

## Highlights

• Angiodysplasias are a frequent cause of gastrointestinal bleeding with a diverse clinical manifestations ranging from symptoms and signs of iron deficiency anemia to occult bleeding and even life-threatening bleeding. This case demonstrates angiodysplasia as an impending source of occult gastrointestinal bleeding.
• According to most authors histological diagnosis of angiodysplasia is based on the presence of mucosal vascular ecstasies with associated prominently dilated capillaries penetrating the muscularis mucosae. In this case these criteria are satisfied; however presence of large, thick walled vessels in submucosa is not described in the classic case of angiodysplasia.
• In this case report: I am discussing this case and making a comparison with the ususal presentation of angiodysplasia, how to diagnose obsure bleeding.
• Role of the radiological as well as triphasic CT scan finding to diagnose the angiodysplasia of the small intestine.
• Incidine of the angiodysplasia in the small intestine.

## 1 Introduction

Angiodysplasia is the second most common vascular abnormality of the GI tract after diverticulosis and is the second principal cause of lower GI bleeding over 60 years [1]. It is considered as degenerative disease that affects the formerly normal blood vessels and found most frequently in ascending colon and cecum in about 75% and 15% are located in the jejunum and ileum, and 10% is disseminated throughout the rest of the alimentary tract. Angiodysplasia is reported as 6% for cases presented by lower GI bleeding [2]. However, the prevalence of GI angiodysplasia in the overall population is not well known. Angiodysplasia has been reported in patients with certain predisposing conditions such as liver disease [3], renal disease [4], von Willebrand disease [5,6] and aortic stenosis [7,8]. The pathogenesis of angiodysplasia remains unclear. Several hypotheses have been anticipated, one of them proposes that the vascular nature of angiodysplasia has a relationship with an elevated levels of the vascular endothelial growth factor (VEGF), resulting in mucosal hypoxia. Another theory postulates that the chronic obstructive consequence at the level of submucosa makes a pressure on the vessels of the submucosa, resulting in its dilatation [9,10]. Grossly: angiodysplasia characteristically are nonpalpable lesions of less than 5 mm in diameter showing tortuous dilation of multiple small submucosal and mucosal blood vessels easier to identify by angiography than in a surgical specimen unless injected with silicone rubber and cleared with methyl salicylate. The work has been reported in line with SCARE criteria [11].

## 2 Case report

**Fig. 1:**
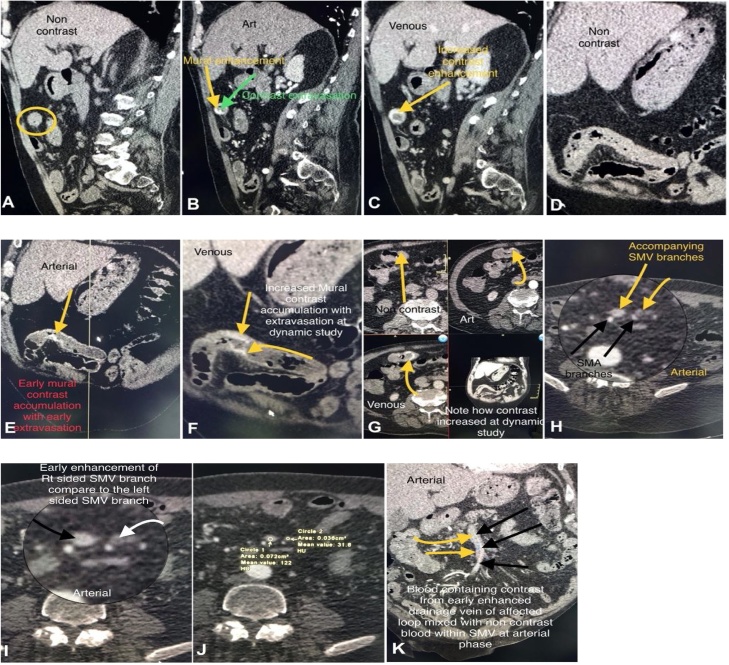
CT abdomen with contrast.

**Fig. 2:**
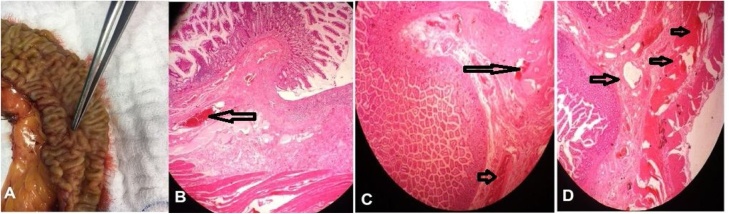
Morphology of angiodysplasia. (A). Gross appearance of angiodsplastic lesion showing tortuous dilation of multiple small submucosal and mucosal blood vessels. (B, C and D) serial histopathological sections showing dilated and thin walled vessels (arrow) (arteries, veins and capillaries) in mucosa and submucosa, the overlying mucosa is eroded and ulcerated as in (C) (H&E stain, x200).

A written consent was taken from patient for publication of the presenting case. A 68-year-old male patient presented to our institute with pallor of gradual onset and progressive course since 6 months. Detailed history revealed hematochezia, sometimes melena. Physical examination revealed signs of iron deficiency anemia which was confirmed by laboratory results and hemoccult positive stool. The patient refused the colonoscopy. A bdominal CT with contrast was then performed and the patient was subjected to preprocedure preparation for 3 days before examination in polyethylene glycol as a bowel-cleansing was administered and low-fiber diet was taken. Additionally, a water-soluble iodinated contrast medium was administered orally for fecal and fluid tagging. CT images were then obtained which revealed the followings: evidence of abnormal subtle mural contrast flocculation within the small bowel loop with luminal extravasations of contrast at small segment of distal ileum 30 cm from ileocaecal valve with mildly dilated and early filling of its drained vein, this abnormal contrast extravasations and flocculation increased through dynamic study. In addition, evidence of contrast mural flocculation within the left lateral wall of distal few centimeters of rectum was also observed (Fig. 1). This abnormal mural contrast flocculation within the wall of solitary segment terminal ileum and distal rectum suggest the evidence of venous ectasia or angiodysplasia. Surgical resection of around 20 cm of the small intestine with end to end anastomosis was done and the resected part sent for histopathological examination which revealed the presence of mucosal vascular ecstasies with associated prominently dilated capillaries penetrating the muscularis mucosae and the overlying mucosa is eroded and ulcerated associated with marked inflammatory cell infiltrates (Fig. 2).

## 3 Discussion

Angiodysplasias are a frequent cause of gastrointestinal bleeding with a diverse clinical manifestations ranging from symptoms and signs of iron deficiency anemia to occult bleeding and even life-threatening bleeding [12]. This case demonstrates angiodysplasia as an impending source of occult gastrointestinal bleeding. Actuality, small bowel angiodysplasias are thought to be responsible for fifty percent of ambiguous, non-variceal gastrointestinal bleeds in adults and the greater part of patients will have several lesions [13].

According to most authors histological diagnosis of angiodysplasia is based on the presence of mucosal vascular ecstasies with associated prominently dilated capillaries penetrating the muscularis mucosae. In this case these criteria are satisfied; however presence of large, thick walled vessels in submucosa is not described in the classic case of angiodysplasia. The differential diagnosis of angiodysplasia includes other varieties of telangectasias, arteriovascular malformations and haemangiomas. Mucosal lymphangiectasia superficially resembles angiodysplasia but the vessels do not contain blood and there is no associated submucosal venous ectasia [14,15].

Angiodysplasia is often segmental or even multifocal, and therefore a careful inspection of the whole bowel should be carried out during the instance of exploration. For lesions found in the right colon which is considered the most common site a right colectomy is highly recommended [16]. There are no reliable recommendations with regard to segmental resections when angiodysplasia is found at other sites, except that for the acute management of severe hemorrhage or for the management of recurrent hemorrhage over a relatively short period accompanied by a large transfusion requirement. In the present case, no further lesions in the immediate area of the ileum or in a different place either in the small bowel or colon were recognized.

Medical therapies including long-acting somatostatin analogues have been shown to diminish the number of re-bleeding on patients with intractable small bowel angiodysplasia [17]. Therapy with argon plasma coagulation via endoscopic procedure is commonly used in due to availability, safety, ease of use, and cost-effectiveness [13]. The fear from occurrence of the complications as bowel infarction associated with use of local infusion of vassopressor drugs as form of super-selective angiographic embolization [18] in addition to its short-lived results as well as age of patient made the use of this treatment plant difficult to attain. Although a large number of primarily efficient therapies are being available in the recent year, the percent of re-bleeding attacks is still high [12].

## 4 Conclusion

This case also emphasizes the critical significance of a mutual multidisciplinary approach to occult gastrointestinal bleeding. The management of angiodysplasia is considered as multidisciplinary team work that would consist of expertise from different departments as in endoscopy, angiography, and gastrointestinal surgery. In addition, council from all these departments should be present at the time of operation when surgery is recommended to optimize the time of finding the difficult lesions and wholly enhanced the chance for achievement.

## Sources of funding

No source of funding and it is my personal effort.

## Ethical approval

The case report is approved from the ethical committee in the college of medicine – Al Baha university.

## Consent

The fully informed written consent is taken from patient.

## Author contribution

I did as a single author the study concept, data collection, data analysis and interpretation, and I wrote the paper.

## Registration of research studies

N/A.

## Guarantor

Dr Thamer Alghamdi.

## Provenance and peer review

Not commissioned, externally peer-reviewed.

## Declaration of Competing Interest

No financial and personal relationships with other people or organisations that could inappropriately influence their work.
